# Micro Elastofluidics: Elasticity and Flexibility for Efficient Microscale Liquid Handling

**DOI:** 10.3390/mi11111004

**Published:** 2020-11-14

**Authors:** Nam-Trung Nguyen

**Affiliations:** Queenland Micro- and Nanotechnology Centre, Griffith University, Nathan, Queensland 4111, Australia; nam-trung.nguyen@griffith.edu.au

Microfluidics is the science and technology around the behaviour of fluid and fluid flow at the microscale [1]. The small size allows processes such as chemical reactions to occur faster and consume fewer reagents. However, the small size also brings phenomena dominated by surface effects that are not encountered at larger scales; this makes storage, mixing, separation and delivery of liquids extremely difficult. One of the major and most successful applications of microfluidics is lab on a chip (LOC), shrinking down a lab-based protocol into a single microfluidic chip. This tool has been particularly useful for many functions in traditional healthcare such as drug delivery, clinical diagnostics, and point-of-care diagnostics. However, all current commercially available LOC platforms, including the more complex organ-on-a-chip (OOC) devices, are single-measurement tools. The recent emergence of wearable and implantable technologies, especially systems that can conform to skin and tissue surfaces, poses new challenges to structural integrity, electronics and fluid handling.

Although progress has been made in the area of materials and flexible electronics [2], microfluidics in wearable and implantable systems remains an almost unexplored area. There are practical and fundamental reasons for the slow progress in this area: (i) current LOCs and OOCs require bulky lab equipment such as syringe pumps and microscopes to operate, and are not practical for wearable devices; (ii) most wearable systems are continuous-measurement tools that require a different design approach than conventional single-measurement LOCs; (iii) storage of samples in vials and syringes, as well as their continuous delivery using pumps, makes them too bulky and not practical for wearable systems; (iv) the flexible and conformal nature of wearable/implantable systems does not allow for the implementation of established methods in conventional rigid microfluidics [1]; and most importantly, (v) the fundamentals of fluid–structure interactions, and the effect of structural flexibility and elasticity on storage, mixing, separation and delivery of liquids in molecular scale and macroscale, are not understood well enough to enable suitable engineering solutions. 

Lack of fundamental knowledge regarding flexibility and elasticity at the molecular scale and the device scale provides a new research area for future discoveries and developments. We recently coined the term flexible microfluidics [3] and formulated the key hypotheses on the effect of device flexibility on microfluidic functions. Micro elastofluidics is the broader research area that covers fundamental phenomena in fluid–structure interactions at the molecular and microscopic scales and enables practical applications in liquid handling and interfacing with biological systems. Discoveries regarding flexibility and elasticity at the interface of microfluidics, electronics and cell biology will provide great opportunities for harnessing the hidden capabilities of future flexible and elastic hybrid systems, well beyond the current state-of-the-art rigid LOC and OOC devices. Figure 1 shows the overview of the concept of micro elastofluidics. Similar to conventional microfluidics, micro elastofluidics can be further divided into digital and continuous-flow micro elastofluidics.

Digital micro elastofludics is based on an individual droplet packaged in a coating of microparticles or an elastic capsule. Packaging a liquid sample into a tiny capsule is not only a potential solution for storage and delivery of liquids on wearable and implantable devices, but also drastically reduces plastic waste in laboratories. State-of-the-art droplet-based microfluidics relies on droplets suspended in an immiscible liquid. The droplets need proprietary and expensive surfactants to remain stable in the suspension liquid, resulting in several limitations. Under well controlled conditions, surfactants can keep the droplets stable. However, heat, mechanical agitation and electric field may cause unwanted coalescence. Loss of sample and contamination are the consequences. Recently, we successfully coated liquid droplets with microparticles to form a liquid marble, an excellent platform for three-dimensional cell culture [4]. However, liquid marbles evaporate quickly if the surrounding humidity is not well controlled. Packaging liquids in an elastic capsule is potentially a solution for storage and delivery of liquid samples. The elastic shell provides mechanical strength to the liquid contents and physically isolates them from the surroundings, thus turning the liquid sample into an elastic solid bead. Existing and future technologies for handling solid particles would then be able to apply to this new platform. We recently demonstrated the feasibility of this platform using the formation of a double emulsion and the subsequent polymerization through exposure to ultraviolet or blue light [5].

While elastic capsules would enable storage and delivery of liquids in a wearable device, novel device concepts of micro elastofluidics for a continuous flow are yet to be discovered and developed. A recent attempt to develop stretchable pumping solution [6] is an example of the vast opportunities yet to be explored. Fundamental understanding of fluid flow in deformable microchannels is needed for designing these microfluidic devices. Furthermore, recent microfluidic devices have emerged for wearable applications, but the effect of fluid–structure interactions in both molecular and device scales has not yet been explored. At the molecular scale, by mixing liquid sample with tiny elastic long-chain polymer molecules to form a viscoelastic fluid, we have observed enhanced mixing [7] and separation [8]. At the device scale, we recently utilised structural elasticity for inertial microfluidics by developing a device with a tuneable channel length [9]. Thus, continuous-flow microelastofluidics has great potential for both wearable and lab-based microfluidic devices.

Flexibility and elasticity provide advantages to conventional microfluidic functions and their interface with biology. Micro elastofluidic hybrid systems are expected to advance state-of-the-art microfluidics beyond the above subfields of digital and continuous-flow micro elastofluidics to connect physics, engineering and life sciences. Most conventional microfluidic devices serve as stand-alone in-vitro platforms to model biological systems, with applications such as organ on a chip. A hybrid system that integrates microfluidics, electronics and the biological system has the potential to bring micro elastofluidics from lab-based to wearable to implantable devices, bridging the gap between artificial and biological systems. Our recent promising results include micro elastofluidics [9], stretchable bioelectronics [10], and cell-stretching-based mechanobiology for early cancer diagnostics [11]. Implantable, micro elastofluidic hybrid systems are of great interest for personalised medicine. The key feature of a micro elastofluidic hybrid system is its capability of continuous monitoring of cellular and tissue activities in real time. The system would also concurrently provide feedback in the forms of chemical, electrical or mechanical stimuli. In this context, the integration of sensing materials with polymeric or elastomeric substrates is of great significance, because these materials allow for the integration of microfluidics, scaffold for cells and conformal attachment to biological tissues.

## Figures and Tables

**Figure 1:**
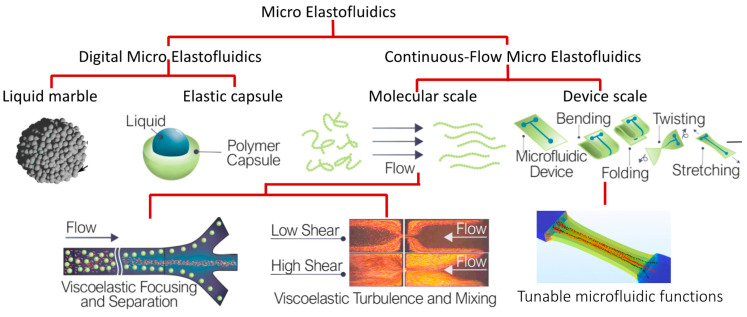
Micro elastofluidics with its possible platforms and applications.
